# The TRUST Principles for digital repositories

**DOI:** 10.1038/s41597-020-0486-7

**Published:** 2020-05-14

**Authors:** Dawei Lin, Jonathan Crabtree, Ingrid Dillo, Robert R. Downs, Rorie Edmunds, David Giaretta, Marisa De Giusti, Hervé L’Hours, Wim Hugo, Reyna Jenkyns, Varsha Khodiyar, Maryann E. Martone, Mustapha Mokrane, Vivek Navale, Jonathan Petters, Barbara Sierman, Dina V. Sokolova, Martina Stockhause, John Westbrook

**Affiliations:** 1 Division of Allergy, Immunology, and Transplantation, National Institute of Allergy and Infectious Diseases, National Institutes of Health, Maryland, USA; 2 HW Odum Institute for Research in Social Science, University of North Carolina at Chapel Hill, North Carolina, USA; 3 Data Archiving and Networked Services (DANS), The Hague, The Netherlands; 4 Center for International Earth Science Information Network (CIESIN), The Earth Institute, Columbia University, New York, USA; 5 World Data System of the International Science Council (WDS), WDS International Programme Office, Tokyo, Japan; 6 PTAB Ltd, Dorset, UK; 7 Universidad Nacional de La Plata, Comisión de Investigaciones Científicas de la Provincia de Buenos Aires, La Plata, Argentina; 8 UK Data Archive, UK Data Service, University of Essex, Colchester, UK; 9 South African Environmental Observation Network, Cape Town, South Africa; 10 Ocean Networks Canada, University of Victoria, Victoria, Canada; 11 Springer Nature, London, UK; 12 University of California, San Diego, California, USA and SciCrunch Inc., San Diego, USA; 13 Center for Information Technology, National Institutes of Health, Maryland, USA; 14 Data Services, University Libraries, Virginia Tech, Virginia, USA; 15 KB National Library of the Netherlands, The Hague, The Netherlands; 16 University Libraries, Columbia University, New York, USA; 17 German Climate Computing Center (DKRZ), Hamburg, Germany; 18 RCSB, Protein Data Bank, Rutgers, The State University of New Jersey, Institute for Quantitative Biomedicine at Rutgers, New Jersey, USA

**Keywords:** Policy, Genetic databases

## Abstract

As information and communication technology has become pervasive in our society, we are increasingly dependent on both digital data and repositories that provide access to and enable the use of such resources. Repositories must earn the trust of the communities they intend to serve and demonstrate that they are reliable and capable of appropriately managing the data they hold.

Following a year-long public discussion and building on existing community consensus^1^, several stakeholders, representing various segments of the digital repository community, have collaboratively developed and endorsed a set of guiding principles to demonstrate digital repository trustworthiness. **T**ransparency, **R**esponsibility, **U**ser focus, **S**ustainability and **T**echnology: the TRUST Principles provide a common framework to facilitate discussion and implementation of best practice in digital preservation by all stakeholders.

## Context and History

For over sixty years, digital data stewardship and preservation have been central to the mission of academic institutions such as libraries, archives, and domain repositories^2^ with many other stakeholders involved, including researchers, funders, infrastructure, and service providers. Scientific data management is receiving increasing attention inside and outside of the scientific community, particularly in the contemporary Open Science discourse. Consensus on ‘good’ data management practice is beginning to form, but there is still insufficient implementation in some scientific domains.

The FAIR Data Principles^3^ highlight the need to embrace good practice by defining essential characteristics of data objects to ensure that data are reusable by humans and machines: they should be **F**indable, **A**ccessible, **I**nteroperable, and **R**eusable, i.e. FAIR. However, to make data FAIR whilst preserving them over time requires trustworthy digital repositories (TDRs) with sustainable governance and organizational frameworks, reliable infrastructure, and comprehensive policies supporting community-agreed practices. TDRs, with their clear remit to actively preserve data in response to changes in both technology and stakeholder requirements, play an important role in maintaining the value of data. They are held in a position of trust by their users as they accept the responsibilities of data stewardship. To fulfill this role, TDRs must demonstrate essential and enduring capabilities necessary to enable access and reuse of data over time for the communities they serve. TDRs support data curation and preservation of data holdings with different levels of reusability. In certain instances, lower-quality data, which cannot reasonably be improved or made more interoperable, may still retain high value to its user community and so require trustworthy stewardship. A TDR must identify and seek to meet community-accepted criteria and communicate the achieved level of data quality.

The *Open Archival Information System (OAIS)* reference model^4^ provides recommendations on setting up archives delivering long-term preservation of and access to information (in particular, digital information) and creating preservation packages. It offers a coherent and comprehensive framework of principles and terminology for the management of archival information systems. However, conforming to the OAIS reference model does not guarantee trustworthiness. In order to assess trustworthiness, additional elements of the repository need to be addressed, including appropriate governance, resources, and security. Furthermore, since OAIS is a reference model and does not provide a detailed implementation guideline, there are different interpretations and implementations necessitating audit and certification mechanisms as recognized in the 1996 report, *Preserving Digital Information*^5^. The authors of the report recommended that “repositories claiming to serve an archival function must be able to demonstrate that they are who they say they are by meeting or exceeding the standards and criteria of an independently-administered program for archival certification”.

Trustworthiness is demonstrated through evidence, which depends on transparency, and thus repositories must provide transparent, honest, and verifiable evidence of their practice. In this way, stakeholders can be confident that repositories ensure data integrity, authenticity, accuracy, reliability, and accessibility over extended time frames. Trustworthiness is not a one-off achievement; it cannot be taken for granted without regular audit and certification.

Certification makes an objective and important contribution to the confidence of the various stakeholders of a repository. To assess and improve the quality of their professional practices, repositories rely on a range of international certification standards covering core, extended or formal level certification. These standards such as the CoreTrustSeal^6^, DIN31644/NESTOR^7^, and ISO16363^8^ focus on four major assessment areas: organization, digital object management, technical infrastructure, and security risk management. The standards vary in the number and complexity of their requirements, with the intensity of assessments ranging from a peer review of a self-assessment to a more involved on-site visit by an external audit team. The choice of certification mechanism depends on the need, willingness, and ability of a repository to invest in its further professionalization and trustworthiness.

The adoption of the CoreTrustSeal Trustworthy Data Repositories Requirements by many data repositories serves as an example of the improvements made to ensure that their capabilities attain the properties of the TRUST Principles^6^. Many data repositories have obtained CoreTrustSeal certification and become members of the International Science Council’s World Data System (WDS). The attainment of certification and the completion of audits by many digital repositories demonstrates the desire for repositories to be perceived as trustworthy.

Repository managers and their teams are the primary audience for the existing OAIS reference model and trustworthiness certification mechanisms discussed above. In an Open Science context, however, we expect that a broader audience, including funders and repository users, will benefit from the framework encapsulated by the TRUST Principles, especially given the increasing attention given to scientific data stewardship (Box 1).

Box 1 The TRUST Principles

**Table 1:** 

**Principle**	**Guidance for repositories**
**T**ransparency	To be transparent about specific repository services and data holdings that are verifiable by publicly accessible evidence.
**R**esponsibility	To be responsible for ensuring the authenticity and integrity of data holdings and for the reliability and persistence of its service.
**U**ser Focus	To ensure that the data management norms and expectations of target user communities are met.
**S**ustainability	To sustain services and preserve data holdings for the long-term.
**T**echnology	To provide infrastructure and capabilities to support secure, persistent, and reliable services.

## Transparency

In order to select the most appropriate repository for a particular use case, all potential users benefit from being able to easily find and access information on the scope, target user community, policies, and capabilities of the data repository. Transparency in these areas offers an opportunity to learn about the repository and consider its suitability for users’ specific requirements, including data deposition, data preservation, and data discovery. To be compliant with this principle, repositories should ensure that, at a minimum, the mission statement and scope of the repository are clearly stated. In addition, the following aspects should be transparently declared:

- Terms of use, both for the repository and for the data holdings.
- Minimum digital preservation timeframe for the data holdings.
- Any pertinent additional features or services, for example the capacity to responsibly steward sensitive data.

Clearly communicating repository policies and, in particular, the terms of use for data holdings, informs users about any limitations that may restrict their use of the data or the repository. Likewise, being able to easily assess whether a repository can handle sensitive data in a responsible manner would also inform their decision on whether to utilize the available data services.

## Responsibility

TRUSTworthy repositories take responsibility for the stewardship of their data holdings and for serving their user community. Responsibility is demonstrated by:

- Adhering to the designated community’s metadata and curation standards, along with providing stewardship of the data holdings e.g. technical validation, documentation, quality control, authenticity protection, and long-term persistence.
- Providing data services e.g. portal and machine interfaces, data download or server-side processing.
- Managing the intellectual property rights of data producers, the protection of sensitive information resources, and the security of the system and its content.

Repository users should have confidence that data depositors are prompted to provide all metadata compliant with the community norms, as this greatly enhances the discoverability and usefulness of the data. Knowing that a repository verifies the integrity of available data and metadata assures potential users that the data holdings are more likely to be interoperable with other relevant datasets. Both depositors and users must have confidence that the data will remain accessible over time, and thus can be cited and referenced in scholarly publications.

Responsibility may be clarified through some legal means (right to preserve) or may take the form of voluntary compliance with some norm (ethical standards).

## User Focus

A TRUSTworthy repository needs to focus on serving its target user community. Each user community likely has differing expectations from their community repositories, depending in part on the community’s maturity regarding data management and sharing. A TRUSTworthy repository is embedded in its target user community’s data practices, and so can respond to evolving community requirements. We take a broad view of ‘user community’ as these could include users depositing or accessing data; those accessing data holdings computationally; and indirect stakeholders such as funders, journal editors, other institutional partners or citizens.

Use and reuse of research data is an integral part of the scientific process, and therefore TRUSTworthy repositories should enable their community to find, explore, and understand their data holdings with regard to potential (re)use. Repositories should encourage users to fully describe data at the time of deposition and facilitate feedback on any issues with the data (e.g. quality or fitness for use) that may become apparent after the data have been made available.

Repositories have a vital role in applying and enforcing the target user community norms and standards as compliance facilitates data interoperability and reusability. Data standards that TRUSTworthy repositories should enforce include metadata schema, data file formats, controlled vocabularies, ontologies, and other semantics where these exist in the user community. A TRUSTworthy repository may demonstrate adherence to this principle by:

- Implementing relevant data metrics and making these available to users.
- Providing (or contributing to) community catalogues to facilitate data discovery.
- Monitoring and identifying evolving community expectations and responding as required to meet these changing needs.

## Sustainability

Ensuring sustainability of a TRUSTworthy repository is necessary to ensure uninterrupted access to its valuable data holdings for current and future user communities. Continued access to data is dependent upon the ability of the repository to provide services over time, and to respond with new or improved services to meet evolving user community requirements.

A TRUSTworthy repository may demonstrate the sustainability of its holdings by:

- Planning sufficiently for risk mitigation, business continuity, disaster recovery, and succession.
- Securing funding to enable ongoing usage and to maintain the desirable properties of the data resources that the repository has been entrusted with preserving and disseminating.
- Providing governance for necessary long-term preservation of data so that data resources remain discoverable, accessible, and usable in the future.

## Technology

A repository depends on the interaction of people, processes, and technologies to support secure, persistent, and reliable services. Its activities and functions are supported by software, hardware, and technical services. Together, these provide the tools to enable the delivery of the TRUST Principles.

A TRUSTworthy repository may demonstrate the fitness of its technological capabilities by:

- Implementing relevant and appropriate standards, tools, and technologies for data management and curation.
- Having plans and mechanisms in place to prevent, detect, and respond to cyber or physical security threats.

## Impact of the TRUST Principles

The TRUST Principles in their abstract, non-technical formulation facilitate communication and thus impact stakeholders both within and outside the data user community. When data repositories, funders, and data creators adopt FAIR Principles and implement the TRUST Principles, repository users benefit directly through continuing and improved capabilities for efficient and effective use of data. Together, the stakeholders of the TRUST Principles contribute to a cultural change in research towards a data and information ecosystem that has been evolving during the information age but has been an essential part of the scientific process for centuries.

Various studies have found that transparency is associated with trust of digital repositories^9^. For example, for users of video data, “transparency of repository practices, and especially data curation practices, are important for trust”^10^. Studying the data repository staff perceptions of repository certification, Donaldson, *et al*.^11^, found that the process of acquiring certification contributed to the transparency of their repository, among other benefits.

The OAIS Reference Model describes the responsibilities of archival information systems that are entrusted with the stewardship of information resources. Describing challenges of effective data stewardship, Peng *et al*.^12^ stated that “Defining roles and responsibilities in every level of stewardship and every stage of the data product lifecycle will help facilitate this challenge”. Furthermore, upon surveying research data practices throughout the data lifecycle, Kowalczyk^13^ reported that “[t]he probability of long-term data management for research collections is low when the ongoing responsibility lies with an individual researcher or graduate student”.

Studying how users’ experiences influenced their perceptions of trust in data repositories, Yoon^14^ found that “users’ awareness of repositories’ roles or functions can be one factor for developing users’ trust”. Users often trust repositories based on their own experiences, repository practices and reputation, and on the experiences of other community members^9,14,15^. Users’ trust in data is also associated with their trust in the archive from which the content was obtained^16^.

The report of a study on the sustainability of digital repositories that was conducted by the Organization for Economic Co-operation and Development (OECD) concluded that “Research data repositories are an essential part of the infrastructure for open science…” [and that it] “is important to ensure the sustainability of research data repositories”^17^. The importance of the sustainability of research data infrastructure has been identified in studies describing the needs of archaeologists^9,18^. In the absence of effective sustainability strategies and continuity plans, data repositories and their holdings could disappear, like many former biological databases^19^. Ironically, York *et al*.^20^ observed that “despite the large number of data repositories, stewardship initiatives, and policies across the research data landscape, we know relatively little about the total amount, characteristics, or sustainability of stewarded research data”.

The adoption of technological capabilities should be completed in conjunction with the organizational, managerial and stewardship capabilities that facilitate the continuing use of a data repository’s holdings^10,21^. Describing the needs for earning public trust of health data, Van Staa *et al*.^22^ called for capabilities that would “combine new technologies with clear accountability, transparent operations, and public trust …”, stating that “data stewardship is not just about physical and digital security: staff training, standard operating procedures, and the skills and attitudes of staff are also important”^22^.

## Conclusions

The TRUST Principles provide a mnemonic to remind data repository stakeholders of the need to develop and maintain the infrastructure to foster continuing stewardship of data and enable future use of their data holdings. The TRUST Principles, however, are not an end in themselves, rather a means to facilitate communication with all stakeholders, providing repositories with guidance to demonstrate transparency, responsibility, user focus, sustainability, and technology.
